# Developing a framework to review near-miss maternal morbidity in India: a structured review and key stakeholder analysis

**DOI:** 10.1186/s12913-014-0553-x

**Published:** 2014-11-13

**Authors:** Sanghita Bhattacharyya, Aradhana Srivastava, Marian Knight

**Affiliations:** Public Health Foundation of India, New Delhi, India; National Perinatal Epidemiology Unit, University of Oxford, Oxford, OX3 7LF UK

**Keywords:** Near-miss, Audit, Quality of care, Framework, India

## Abstract

**Background:**

In India there is a thrust towards promoting institutional delivery, resulting in problems of overcrowding and compromise to quality of care. Review of near-miss obstetric events has been suggested to be useful to investigate health system functioning, complementing maternal death reviews. The aim of this project was to identify the key elements required for a near-miss review programme for India.

**Methods:**

A structured review was conducted to identify methods used in assessing near-miss cases. The findings of the structured review were used to develop a suggested framework for conducting near-miss reviews in India. A pool of experts in near-miss review methods in low and middle income countries (LMICs) was identified for vetting the framework developed. Opinions were sought about the feasibility of implementing near-miss reviews in India, the processes to be followed, factors that made implementation successful and the associated challenges. A draft of the framework was revised based on the experts’ opinions.

**Results:**

Five broad methods of near-miss case review/audit were identified: Facility-based near-miss case review, confidential enquiries, criterion-based clinical audit, structured case review (South African Model) and home-based interviews. The opinion of the 11 stakeholders highlighted that the methods that a facility adopts should depend on the type and number of cases the facility handles, availability and maintenance of a good documentation system, and local leadership and commitment of staff. A proposed framework for conducting near-miss reviews was developed that included a combination of criterion-based clinical audit and near-miss review methods.

**Conclusion:**

The approach allowed for development of a framework for researchers and planners seeking to improve quality of maternal care not only at the facility level but also beyond, encompassing community health workers and referral. Further work is needed to evaluate the implementation of this framework to determine its efficacy in improving the quality of care and hence maternal and perinatal morbidity and mortality.

## Background

One of the key indicators used to assess maternal health is the maternal mortality ratio and, further to this, review of maternal deaths has been widely recommended [1]. Review of nearmiss obstetric events has been suggested to be additionally useful to investigate health system functioning, particularly as maternal death becomes less common [2]. An estimated 289 000 maternal deaths occurred in 2013, a decline of 45% from 1990, but 300 million women suffer from long-term or short-term illness due to pregnancy or childbirth [3,4]. As maternal deaths are relatively rare events, in order to overcome the difficulty in estimation and to track quality of service delivery, examining near-miss events has the potential to complement the maternal death reviews [5]. Thus strengthening the health system and providing optimal care for women during pregnancy and childbirth is imperative not only to prevent deaths but also crucial to avert acute pregnancy-related complications and disabilities [6,7].

Audit of maternal deaths is considered a comprehensive way of checking the functioning of the health facility and also to identify gaps in service provision through use of a set of process indicators [8]. Similarly, assessment of near-miss cases is seen as a useful method, complementing maternal death reviews, and can provide a monitoring tool for quality and performance of obstetric services [9,10]. Compared to maternal death review, the fear of staff blame and punishment is less in near-miss cases. Thus, if the review is done effectively, it can lead to improvement in quality of services which may not occur when fear leads to poor participation in maternal death reviews [11-13]. Even in resource-poor settings, the number of maternal deaths per facility or in a region may not allow detailed quantification of risk factors and determinants which are of local significance [12,13]. As near-misses occur much more frequently than maternal deaths, more statistically reliable quantitative analyses can be achieved. These provide a comprehensive profile of the health system functioning and assist in developing coordinated actions to address the identified barriers to quality care [14-19].

In India, recently introduced health sector reforms have managed to increase the demand for institutional delivery, resulting in an unprecedented growth in the rate of institutional births. Janani Suraksha Yojna (JSY), a conditional cash transfer scheme, has been instrumental in encouraging women to deliver in health facilities. Institutional deliveries in India have expanded from 53% in 2005 to 73% in 2009–10 [20,21]. This phenomenal increase has also brought to light the problems of overcrowding, with resultant compromises in quality of care. Decline in the standard of care may act as a deterrent, as women and communities recognize shortfalls in safe and respectful care. The Government of India has recommended community based maternal death review (CBMDR) and facility based maternal death review (FBMDR) to help in identifying the gaps in the existing health care delivery systems, prioritize and plan for intervention strategies to prevent maternal death and reconfigure health services [22]. A maternal death reporting form is already part of the Health Management Information System (HMIS) and health facility staff and program managers have received orientation on how to review them and take decisions based on them. There are no recommendations currently for undertaking near-miss reviews, which would complement the existing maternal death review.

The aim of this project was to identify the key elements required for a near-miss review programme through a literature analysis, and to conduct a key stakeholder analysis to develop a comprehensive framework for India, integrating the different levels of care in order to understand and identify actions to address the system deficiencies.

## Methods

A structured review was conducted to identify the various methodologies used in assessing near-miss cases. Google scholar, PUBMED, MEDLINE, Popline and the World Health Organization Global Health Library were searched using the key letters ‘maternal morbidity, near-miss, maternal death’, ‘maternal audit’, ‘near miss review’. The review was restricted to techniques used in LMICs. In addition, the website of the Indian health ministry was also searched for policy documents on guidelines or methods currently being promoted or used in India to assess maternal death and morbidity. The methodologies used were assessed based on the setting in which they were undertaken (i.e. whether at facility or community levels); the stakeholders involved in conducting the review; the types of tools used and the strengths and weaknesses of the methods. The findings of the structured review were used to develop a suggested framework and options for conducting near-miss reviews in India.

Based on the structured review, a pool of experts who have worked extensively in either testing any of the near-miss review methods or validation of frameworks in LMICs was identified for vetting the framework. Eleven experts were identified, five from India and six who have worked in other LMICs such as sub-saharan and west African countries, Caribbean and Latin American countries. Experts included senior public health researchers, programme managers and technical experts. A guide highlighting the key themes for consultation was developed based on the structured review findings and the draft framework (Table 1). Discussions were held both in-person and by telephone, as per the convenience of the respondent. Written notes of the responses were taken.

**Table 1:** **Themes for consultation with experts**

**Theme/sub-themes**	**Details**
**1. Experience on implementing a method to assess near-miss**	
Background	Geographical area in which near-miss review was tested/implemented; number and type of women/institutions covered; duration of implementation
Review design	Conceptual framework on which the process was designed; tools and techniques used
Process and outcomes	Details of implementation; highlights of findings; success achieved and challenges
Sustainability	Discussion on sustainability of such a process of near-miss assessment in a LMICs context
**2. Replicability and relevance of the approach**	
Relevance	Applicability in LMICs and Indian context
Modifications, if any	Any modifications required to adapt the method to LMICs context
**3. Future course of work**	
Implementation process in India	Steps in initiating near-miss review in Indian context; challenges and sustainability of the approach
References	Other experts/groups working on near-miss in India or other LMICs; any references of published or unpublished research on the theme

Opinions were sought from the experts about the feasibility of implementing near-miss reviews in India, the processes to be followed, factors that make implementation successful and the associated challenges. Their views were sought on the framework developed on the basis of the structured review and its relevance in the present context. The discussion covered all the themes of the consultation guide and interviews continued until no new suggestions emerged on how to develop a framework to assess near-miss. A snowball technique was used to identify experts until thematic saturation was reached. Notes from the interviews were analysed thematically based on the elements that needed to be considered while undertaking a near miss review in LMICs. The draft framework was revised based on the analysis of the interviews.

This study adheres to the RATS qualitative research guidelines and has been granted exemption from ethical review by the institutional ethics committee of the Public Health Foundation of India (TRC-IEC-222/14).

## Results

The structured review identified five broad methods to conduct review and audit of near-miss cases or severe maternal morbidity in LMICs (Table 2).

**Table 2:** **Summary of methods to conduct near–miss assessment**

**Methods**	**Approach**
Near-miss case review	An in-depth understanding particularly of the entire process of care conducted at the local level that includes administrative, managerial aspects, as well as the opinion of the patient about the care she received. Such reviews can identify the combination of factors at the facility and in the community that contributed to near-miss cases. There are three aspects to conducting case reviews of near-miss cases: First using the “gate to gate” approach from admission to discharge, a case may be observed throughout her stay. The method can help to identify the physical locations of any delay and also during what time period it occurred. Secondly, analysis of a sample of cases based on the medical records to understand the overall functioning of the health facility and the gaps in providing care. Thirdly, interviews with women who had near-miss events to understand the woman’s account of her care [17,19]. The method provides a holistic assessment of the health system from both the demand and supply side and also the issue of quality improvement can be looked at from a multi-disciplinary approach, bringing clinical and non-clinical staff together in a common forum. The patient’s perspective gives the opportunity for survivors to share their experience and views. The main constraint is sustainability as the process requires a significant amount of time and resource and there are challenges in embedding it as part of a routine surveillance system [11,17].
Confidential enquiries into near-miss cases	An anonymous investigation of a representative sample of near-miss cases. Reviewing of data by an independent expert panel helps to identify causes and avoidable or remediable factors associated with near-miss morbidity [23,24]. The method allows a group to understand the factors contributing to poor outcomes and to learn lessons for the future by assuring confidentiality and not reporting details of individual cases thus preventing punitive action [11,24].
Criterion-based clinical audit	In this method prior agreement is reached of a list of concise criteria for good quality care, based on available evidence and resources. All records of women are reviewed to determine the care received against these explicit criteria. It is used as a part of the quality improvement process to improve patient care and outcomes through systematic review of aspects of the structure, processes, and outcomes of care [25,26]. The key strength of the method is that data can be obtained retrospectively from case notes or registers, it is easy to interpret and the quality of care based on agreed criteria of best practice can be assessed. The main challenge is that retrospective collection of information might be problematic due to poor documentation [27].
Structured review (South African Model)	This method involves review of all cases of severe morbidity focusing on three main areas: patient-related problems, administrative problems and clinical care [12]. The method is useful as care can be assessed over six distinct periods; antenatal, intrapartum and postnatal care, and three phases related to emergency care;admission, resuscitation and anaesthesia. The method also helps to identify barriers at both patient-related as well as health systems levels [28].
Home-based interviews	Women are interviewed at home within one month of discharge. Two groups of women may be interviewed: those who are identified as part of facility near-miss reviews and women who have not been identified by near-miss reviews [28]. The method provides the perspective of the user of services and can identify delays at family and community level. The main challenge is women’s willingness to share experience with health system representatives. Moreover a woman may not be able to narrate her whole experience due to her physical state. Interviews with women needs trained data collectors and time and resources in locating the women for interview and analysing the data.

### Expert perspectives on key considerations for near-miss reviews

The perspective of the experts on the key elements needed for a facility to undertake a near-miss review can be classified under four broad headings: identifying the appropriate method; adopting a step by step approach; not limiting the review process to an audit and engaging personnel at different levels in the health system.

#### Identifying the appropriate method

The structured review identified methodological options for near-miss reviews, such as criteria-based clinical audit and case reviews. However, the experts recognized that near-miss reviews involve time and resources in the facility. Therefore they felt the method to be adopted by a facility should be identified after assessing some key implementation aspects. The experts highlighted three considerations based on which the review method could be decided.

Firstly, the number and type of cases the facility handles, i.e. whether it is primary care only, a referral centre at the secondary level or a tertiary care centre. This will determine the volume of near-miss cases the facility receives. Accordingly an individual centre can decide on whether the near-miss reviews and audit would involve review of all near-miss cases or review of a subset, such as the audit of all cases of a particular condition.

Secondly, availability and maintenance of good data and documentation system was noted to be a deciding factor as it will determine whether there is need for introduction of new data collection formats for capturing the details of near miss cases, depending on the particular method being considered.

Finally, policy level commitment and particularly local leadership and commitment of the staff was thought to be critical for sustainability of any approach or method for near-miss review.

#### A step-by-step approach

The experts felt that initiating a review and sustaining it should be a key objective of the facility. Therefore the decision on the method to be followed for near-miss review should be made through a collective process, which could involve both clinical and managerial staff to maximize ownership. This was thought to also help familiarize them with the process, assess the gaps in their data and documentation processes and ultimately allow the findings of the review to be linked with their own quality improvement plans. The respondents further emphasized that the frequency of the review process also needed to be decided jointly amongst all staff, and should be realistic and doable.

#### Review processes in addition to an audit

Maternal death and severe morbidity audit can be a good quality marker. Quality improvement programs can use information about causes of deaths and morbidity to understand substandard care and how it can be averted. Respondents felt that near-miss case assessment provided further direction on how to improve the care that is provided, particularly where explicit audit standards do not exist. The changes identified as part of near-miss case reviews may be implementable at individual, team, or service level and, with further monitoring, can improve healthcare delivery. The experts felt that near-miss assessment can an effective tool to highlight the deficiencies as well as the positive elements in the provision of obstetric services in any health system and thus can be a good standard for any quality improvement process.

#### Need for a coordinated approach engaging personnel at different health system levels

Most of the experts felt that near-miss assessment should not be restricted to secondary or tertiary care levels. To understand a composite picture of the whole system (in a district), particularly the factors leading to delay in seeking and accessing care responsible for the near-miss condition of the women, it was felt to be additionally helpful to understand the factors at the community and primary care levels. Facilities conducting a near-miss review could therefore link with other facilities comprising the referral chain in their region.

### A comprehensive framework to understand the system gaps at primary level in India

**Figure 1:**
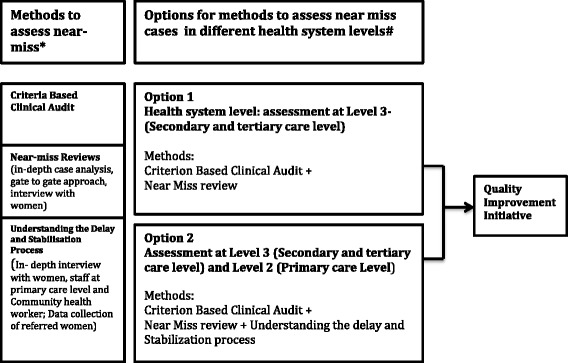
**Methods and options to assess near-miss cases.** *Existing methods to conduct near-miss assessment based on structured review. #Options identified based on stakeholders consultation.

Based on the structured review and expert perspectives, a comprehensive framework for assessing maternal near-miss was developed. The framework provides options for health facilities to adapt and use either one or multiple methods of near-miss assessment, in order to monitor and improve their quality of maternal health services [Figure 1]. Option 1 is suitable for a large secondary/tertiary level facility handling complicated deliveries and acting as a referral hospital. It includes both criteria-based clinical audit and near-miss review. By conducting both audit and near-miss review the facility can identify gaps in its functioning, identify bottlenecks and the findings can feed into its quality assurance program. To conduct an audit, cases can be found both prospectively and retrospectively from the admissions and discharge registers, case notes and operating theatre book. It is important that a systematic approach is used to search these sources to avoid either missing or double-counting cases. Staff at the facilities can decide the cases that are to be audited and case information to be extracted by trained personnel (can be non-clinical staff) at the health facility. During data extraction, guidance from senior staff is advisable to ensure data quality. Criteria can be based on national or WHO guidelines. The facility can adapt these criteria for local use. While developing the criteria it must be taken into consideration that they are essential rather than optional and should be auditable from existing patient records. The frequency of data collection can vary based on the volume of deliveries that happen at the health facility and types of cases the facility receives, but it is advisable to undertake an audit at least twice a year. It is advisable to undertake random crosschecks of the data, with double data entry of at least 5% of cases. Based on what resources are available, the quality of patient records and type and volume of cases that are handled by the facility, cases can be selected either on the basis of clinical features such as hemorrhage, infections, eclampsia or organ dysfunction, or management criteria such as admission to ICU, need for blood transfusion etc.

In conducting case reviews, a sample of near-miss cases can be identified and discussed by clinical and administrative staff at facility. The review team from the facility can prepare a case history, present the findings and facilitate the discussion in the meeting. The case history should include a woman’s age, obstetric history, condition during arrival, initial diagnosis, main procedure and other treatment provided, level of staff providing care, and outcome of care. The review should highlight favourable management procedures that saved the life of the woman, as well as adverse factors that led to the near-miss situation.

**Figure 2:**
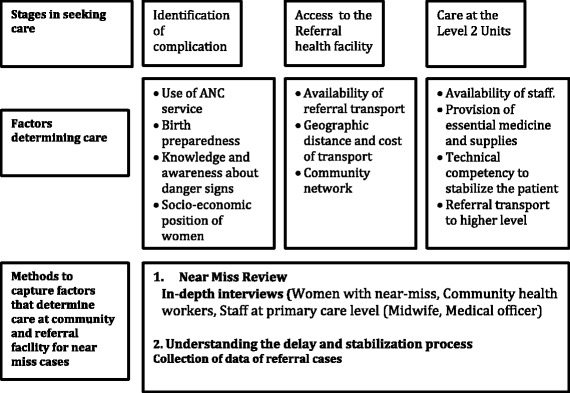
**Framework to assess gaps for near-miss cases at level 2 (Primary care level).**

Option 2 (Figure 1) is a more holistic approach where a secondary/tertiary level facility and its referral areas can be considered as a single unit where near-miss reviews can be conducted. This will not only assess the care provided at the secondary level but can also identify delays at the lower level facilities as well as in the community. If the health facility opts for option 2, the framework below [Figure 2] suggests the factors beyond clinical care which could be examined, including birth preparedness, knowledge of women/family members to allow them to identify danger signs and availability of transport. In order to understand the delays at this level a few in- depth interviews with the community health workers and staff at the Level 2 facilities can be conducted by the case review team constituted at the Level 3 facilities. Community health workers are the first point of contact in the health system and are responsible for providing antenatal care, providing counselling to identify danger signs, arranging transport particularly during emergencies and accompanying women to immediate and higher level facilities. Interviewing them will help to assess the gaps, particularly in birth preparedness, transport facilities and delay in the whole referral chain. The competency of the staff at primary care level and availability of essential medicines and supplies to stabilize women before referring them to higher-level facility are determining factors which could be examined by extracting information from facility records. The main aim is to understand the stabilization process that facilities are conducting before referring woman to higher-level facilities. This will help to identify gaps in supplies, essential medicine and clinical competency of the staff at the facility. In-depth interviews with women who have had a near-miss can help to identify solutions to reduce delays in accessing care. To ensure sustainability of the approach, these interviews can be conducted by a public health manager or a member of staff who is part of the case review team at the facility. Preferably interviews should not be conducted by someone who was involved in the direct care of the woman.

In order to have ownership of the findings and to devise solutions, the team constituting the assessment of near-miss cases at both the levels of the health system should be from the facility, particularly staff who attended the women, administrators of the health system and quality assurance program managers. This will ensure there is a holistic approach in analyzing gaps and deficiencies in the facility and devising solutions.

## Discussion

This review and stakeholder analysis highlighted different approaches and methods that a health facility can adopt to identify and assess near-miss cases. The methods that a facility adopts depend on the type and number of cases the facility handles, availability and maintenance of good data and documentation system, availability of resources and local leadership and commitment of the staff. The proposed framework provides a holistic view of near-miss assessment and review if implemented in a geographical area by facilities at different levels. The inclusion of facilities at different levels can provide information about the overall functioning of the health system, thus implementing quality of care interventions in a more comprehensive way.

Over the last two decades lessons have been learned concerning various methods to conduct maternal death review; some of the approaches have also been tested to assess near–miss or other maternal morbidity [5,10,11]. The methods include reviewing all the maternity records to determine whether the care received meets a pre-agreed set of criteria of care [5,13,25], following the women from admission to discharge [14,17], interviews with women or family members of women who experienced near-miss to learn about the care she received [19], and in-depth analysis of selected cases by an independent expert panel [14], Ministry of Health and Family Welfare: Maternal near miss in India, a report on developing maternal near miss policy framework, definition, criteria and tools, forthcoming]. Recently the Government of India’s Ministry of Health appointed an expert committee to develop criteria for ‘Maternal Near Miss’ (MNM). This committee developed criteria to identify near-miss cases and tools to conduct audits at the tertiary facility level. An audit enables the facility to identify the factors that contributed to a near-miss and determine whether it is linked with a health problem identified during the antenatal period, or detection and handling of the case at the facility [Ministry of Health and Family Welfare: Maternal near miss in India, a report on developing maternal near miss policy framework, definition, criteria and tools, forthcoming].

In low resource settings, it is often seen that many women reach higher-level health facilities in very poor health condition. This is often attributed to the delay in recognition of danger signs by the women and family members themselves, or delay in accessing the first level of care and further referral to the higher-level facilities [23,28]. The average interval from onset of a major obstetric complication, such as post-partum hemorrhage, to death ranges from 2–5 hours, so any delay in the system can worsen a woman’s condition. In spite of good care that may be available at the higher level health facility, she could suffer from serious complications which may lead to her death [28]. To develop a composite picture of maternal care available in a district (the basic sub-regional administrative unit in India), particularly the factors leading to the near-miss condition of women, it is imperative to understand the factors at the community and primary care levels.

The framework highlighted in the study moves beyond clinical factors to address the issue of care provided by community level workers in terms of birth preparedness, knowledge of women/family members to identify danger signs and availability of transport, which plays a key role in accessing health services. One of the significant tools for assessing near-miss cases is the narrative of the survivor, which is critical for identifying deficiency in care. The survivor’s account can identify sub-standard care and assess the barriers at both the primary and secondary care level [19,24]. Similarly the community health workers are the first point of contact in the health system and are responsible for providing antenatal care and counseling to help identify danger signs, arranging transport, particularly during emergencies, and accompanying women to immediate and higher level facilities. Interviewing community health workers could help to assess gaps, particularly in birth preparedness, transport links and other delays in the referral chain. As the causes of near-miss cases are often attributed to the delay in seeking and accessing care [17,24,26,28-30], in order to capture the delay at primary care levels, a combination of different approaches encompassing the both the primary and secondary levels seems the optimal solution [28,31,32].

## Conclusion

This paper proposes a comprehensive framework for assessing maternal near-miss in the Indian context, where access to and quality of primary care are likely to play important roles in precipitating maternal complications. The framework has been developed as a potential guide for researchers and planners seeking to improve overall maternal quality of care, not only at the facility level but also beyond, encompassing community health workers and referral. This framework, in terms of approach and tools for a comprehensive near-miss assessment involving both secondary and primary care level, can be integrated with the audit criteria developed by the Ministry of Health in India to assess near-miss cases [Ministry of Health and Family Welfare: Maternal near miss in India, a report on developing maternal near miss policy framework, definition, criteria and tools, forthcoming]. Further work is needed to evaluate the implementation of this framework, in terms of barriers to implementation, costs and outcomes to determine its efficacy in improving the quality of care and hence maternal and perinatal morbidity and mortality.

## Abbreviations

JSY: Janani Suraksha Yojna
CBDMR: Community based maternal death review
FBDMR: Facility based maternal death review
HMIS: Health Management Information System
